# Inhibitory activity of chokeberry, bilberry, raspberry and cranberry polyphenol-rich extract towards adipogenesis and oxidative stress in differentiated 3T3-L1 adipose cells

**DOI:** 10.1371/journal.pone.0188583

**Published:** 2017-11-28

**Authors:** Katarzyna Kowalska, Anna Olejnik, Dominik Szwajgier, Mariola Olkowicz

**Affiliations:** 1 Department of Biotechnology and Food Microbiology, Poznań University of Life Sciences, Poznań, Poland; 2 Department of Biotechnology, Human Nutrition and Science of Food Commodities, University of Life Sciences, Lublin, Poland; Universita degli Studi di Catania, ITALY

## Abstract

Berries are a rich source of antioxidants and phytochemicals that have received considerable interest for their possible relations to human health. In this study, the anti-adipogenic effect of polyphenol-rich extract obtained from chokeberry *Aronia melanocarpa* (Michx.) Elliot, raspberry *Rubus idaeus* L., bilberry *Vaccinium myrtillus* L. and cranberry *Vaccinium macrocarpon* Aiton fruits and its underlying molecular mechanisms were investigated in differentiated 3T3-L1 adipose cells. Treatment with the extract (25–100 μg/mL) significantly decreased lipid accumulation and reactive oxygen species generation in adipocytes without showing cytotoxicity. Real-time PCR analysis revealed that the extract at a concentration of 100 μg/mL suppressed adipogenesis and lipogenesis via the down-regulation of *PPARγ* (67%), *C/EBPα* (72%), *SREBP1* (62%), *aP2* (24%), *FAS* (32%), *LPL* (40%), *HSL* (39%), and *PLIN1* (32%) gene expression. Moreover, the extract significantly increased the expression of adiponectin (4.4-fold) and decreased leptin expression (90%) and respectively regulated the production of these adipokines in 3T3-L1 adipocytes. The obtained results suggest that the analyzed extract may be a promising source of bioactive compounds that support long-term weight maintenance and promote the effective management of obesity.

## Introduction

Obesity and concomitant metabolic complications have become global health problems with increasing prevalence that affect both genders, every ethnicity and all ages. Food and nutrition play a key role in the prevention and treatment of obesity and obesity-related metabolic disorders such as cardiovascular disease, hypertension, diabetes, and dyslipidemia [1]. Obesity management through diet can be achieved by identifying bioactive functional food ingredients that could modulate molecular pathways and gene/protein expression in a beneficial way [2]. Currently, the only validated therapeutic measure consists of preventing hypertrophy in adipocytes via caloric restriction or increased caloric expenditure. Food-derived polyphenols may evoke effects supporting strict calorie diet by synergistic interactions with multiple targets [3]. Increasing evidence suggests that the health benefits of plant foods are attributed to the synergy or interactions of bioactive compounds and other nutrients in whole foods [4]. Because of the limited efficiency and the side effects of the drugs that are available for treating obesity, natural bioactive plant-derived materials are a promising source for functional food product development to support long-term weight maintenance and promote the effective management of obesity and other metabolic complications [2, 4].

Numerous recent studies have indicated that berries rich in polyphenols such as phenolic acids, flavonoids (anthocyanins and flavonols) and tannins [5], can provide great advantages in preventing or mitigating metabolic syndrome components, including obesity, type II diabetes, and lipid disorders [6]. The anti-obesity mechanisms of berries may include a reduction in lipid absorption, a decrease in differentiation and proliferation of preadipocytes, a decrease in lipogenesis, an increase in lipolysis and the inhibition of pro-inflammatory adipokine secretion [7–10].

The aim of this study was to investigate the in vitro effects of the multicomponent polyphenol extract prepared from chokeberry, raspberry, bilberry and cranberry fruits on the regulation of lipogenesis and adipogenesis at both the molecular and cellular levels. Because of the important biological roles of adiponectin and leptin and their significance to obesity and obesity-related diseases, the effects of the berry fruit extract on the secretion of these adipokines were also evaluated.

## Materials and methods

### Preparation of berry fruit extract

Chokeberry *Aronia melanocarpa* (Michx.) Elliot and raspberry *Rubus idaeus* L. were cultivated on a farm located at 66A Kopernika Street, Bełżyce, Poland (N 51.17608°, E 22.26770°). Bilberry *Vaccinium myrtillus* L. and cranberry *Vaccinium macrocarpon* Aiton were obtained from the Partnership Wholesale Market S.A. located at 65 Elizówka Street, Ciecierzyn, Poland (N 51.287978°, E 22.580237°). Fruit samples weighing 1000 *g* each were homogenized using a Thermomix TM31 food processor (Vorwerk, Wuppertal, Germany). The fruit slurry was then extracted with water and centrifuged (30 min, 9000 *g*, 4°C). The solid residue after centrifugation was subjected to re-homogenization, centrifuged and once more extracted to yield in total approximately 3000 mL of crude extract.

The combined solutions from the extracted samples were then diluted with deionized water (4:1 ratio) and filtered using the VivaFlow 50 filtration system (Sartorius AG, Goettingen, Germany) through 0.22 μm pore-size polyethersulfone (PES) and 5000 MWCO PES membranes, respectively. After ultrafiltration, the extract was vacuum concentrated at 35°C (Büchi, Switzerland).

### Preparative HPLC

The vacuum concentrated samples from each fruit underwent HPLC purification in a BioLogic DuoFlow system (Bio–Rad, USA) on a preparative 250 mm × 20 mm i.d. Eurospher 100–5 C_18_ column (Knauer, Berlin, Germany). Different chromatographic elution programs and mobile phases were tested to remove nonphenolics (mainly sugars and organic acids) from the preparation. The final mobile phase solvents were: (A) ultrapure water and (B) 96% ethanol, and the elution protocol (B in A) was as follows: 0–10 min, 0% B; 10–15 min, 0–90% B; 15–30 min, 90% B; 30–35 min, 90–0% B, followed by 7 min equilibrium time. The column temperature was set to 16°C, and the flow rate was 10.0 mL/min. Compounds were detected at wavelengths of 245 nm, 280 nm, 365 nm and 530 nm. All signals were analyzed using the BioLogic DuoFlow V.5.10 Build 2 software. Fractions obtained after the separation were investigated for the presence of reducing sugars and phenolic compounds; those without phenolics were discarded.

The separation was repeated dozens of times and fractions containing phenolic compounds were combined and concentrated under vacuum (35°C). The extract from each fruit was standardized to obtain 40 mg of total phenolic compounds/mL. The final berry fruit extract (BFE) analyzed in this work was prepared by mixing individual fruit preparations in equal volumes.

### HPLC/DAD/ESI-MS^n^ analysis

Samples were analyzed on an Agilent 1200 series HPLC system (Agilent Technologies, Inc., Santa Clara, CA, USA) comprising a G1312A binary pump, a G1315D photodiode array detector scanning from 190 to 600 nm, and a G1329 autosampler cooled to 4°C. Chromatographic separations were carried out on a 150 mm × 2.1 mm, 5 μm ACE (Advanced Chromatography Technologies, Aberdeen, Scotland) C_18_ column that was maintained at 25°C. The mobile phase consisted of two solvents: formic acid (5%, v/v) in water (A) and methanol (B). A linear gradient starting with 5% B, 5% B at 1 min was set to reach 25% B at 8 min, 45% B at 30 min and 95% B at 40 min. The flow rate was 0.3 mL/min, and the injection volume was 5 μL. The HPLC chromatograms were recorded at 280 nm for flavan-3-ols, at 325 nm for conjugated forms of hydroxycinnamic acids, at 360 nm for flavonols and at 520 nm for anthocyanins.

After passing through the flow cell of the DAD detector, the column eluate was directed to an Agilent 6224 time-of-flight MS system fitted with electrospray ionization (ESI) source. The mass spectrometer was operated in either positive or negative ionization mode, depending on the physicochemical properties of the compounds used. Major mass spectrometer parameters were as described previously. Instrument control, data collection, and analysis were achieved with the MassHunter 2.0 software (Agilent Technologies, Inc.). Phenolic compounds in samples were identified by matching their spectral (UV-Vis or MS) characteristics against those of standards or derived from published data.

For quantification purposes, all phenolic acids were expressed as 4-hydroxybenzoic acid, 3,4-dihydroxybenzoic acid (protocatechuic acid), 3,4,5-trihydroxybenzoic acid (gallic acid) or 3-*O*-caffeoylquinic acid (chlorogenic acid) equivalents; all flavan-3-ols and their polymers as (-)-epicatechin equivalents; anthocyanins conjugates as cyanidin-3-*O*-galactoside equivalents, and flavonols conjugates as quercetin-3-*O*-galactoside equivalents. All standards were purchased from Sigma–Aldrich (Steinheim, Germany).

### 3T3-L1 cell differentiation

The mouse embryo 3T3-L1 cell line was obtained from the American Type Culture Collection (ATCC, CL-173). The 3T3-L1 cells were grown in Dulbecco’s Modified Eagle’s Medium (DMEM) (Sigma–Aldrich) that was supplemented with 10% calf serum (Sigma–Aldrich). For adipocyte differentiation, the 3T3-L1 cells were grown in 24-well plates inoculated at 2.5 × 10^4^ cell/cm^2^ to confluence. Two days post-confluence, preadipocytes were stimulated by a differentiation mixture containing 0.25 μM dexamethasone (DEX), 0.5 mM 3-isobutyl-1-methylxanthine (IBMX) and 1 μM insulin (Sigma–Aldrich) in DMEM with 10% fetal bovine serum (FBS) (Gibco, Life Technologies Carlsbad, CA, USA). After 2 days, the medium was replaced with DMEM containing 10% FBS and 1 μM insulin. Cultures were incubated for 2 days, after which the culture medium was replaced with DMEM/10% FBS and refreshed at 2-day intervals thereafter, until the analysis was performed on days 6–8. For the experiments, BFE at a concentration of 25, 50 and 100 μg/mL was added to the medium at each of the three stages of the differentiation process.

### Adipocyte cell viability assay

The viability and metabolic activity of differentiated adipocytes were determined using the MTT (3-(4,5-dimethylthiazol-2-yl)-2,5-diphenyltetrazolium bromide) assay (Sigma–Aldrich) as described previously [11].

### Determination of reactive oxygen species in adipocytes

The level of intracellular reactive oxygen species (ROS) was determined using nitro blue tetrazolium (NBT) according to the procedure described by Choi [12]. The cells were incubated in 0.2% NBT solution for 90 min, washed with phosphate-buffered saline and methanol, and then air-dried. The formazan was extracted from the cells with 2 M KOH and dissolved in DMSO. The absorbance was measured at a wavelength of 620 nm using a Tecan M200 Infinite microplate reader (Tecan Group Ltd., Männedorf, Switzerland).

### Determination of lipid accumulation by Oil Red O staining

The lipid content in the mature adipocytes was determined using the Oil Red O staining method. The cells were fixed with 10% formalin for 1 h, washed with 60% isopropanol, and completely dried. Then, the cells were stained with Oil Red O (Sigma–Aldrich) solution in isopropanol for 10 min and washed four times with water. Fat droplets stained red were extracted from cells using isopropanol, and the absorbance was measured at a wavelength of 500 nm (Tecan M200 Infinite).

### Quantification of gene expression using real-time PCR

Total RNA was isolated from adipocytes using TRI-Reagent (Sigma–Aldrich) according to the manufacturer’s instructions. First-strand cDNA synthesis was performed with 1 μg of total RNA using a Transcriptor First Strand cDNA Synthesis kit (Roche Diagnostics GmbH, Mannheim, Germany), following the manufacturer’s protocol. The resulting cDNA was amplified using a real-time quantitative PCR system (SmartCycler DX real-time PCR System Cepheid, USA) with SYBR^®^ Select Master Mix (Life Technologies). PCR was performed in a final volume of 25 μL, including 10 ng of sample cDNA, 5 μM of specific forward and reverse primers, and 12.5 μL of SYBR^®^ Select Master Mix. The primers used for the amplification of cDNAs are listed in S1 Table. The reaction mixtures were incubated for an initial denaturation at 94°C for 10 min, followed by 40 PCR cycles: 40 s at 95°C, 30 s at 59°C and 30 s at 72°C. The purity of the PCR products was determined on the basis of melting curve analysis. The relative amount of each gene was calculated using the 2^-ΔΔCT^ method [13]. The levels of each different mRNA in the control cells were designated 1, and the relative levels of the gene transcripts in the samples were expressed as the fold change. A value of <1 indicates transcriptional down-regulation (inhibition of gene expression) compared with control cells that were not treated with BFE. All reactions were performed in triplicate.

### Measurement of adipokine production

The leptin and adiponectin concentrations were measured using sandwich ELISA kits (Millipore, Merck Group, Darmstadt, Germany) following the manufacturer’s instructions. The adipokine concentrations were expressed as ng/ml of culture medium, which was equivalent to the amount of protein per 1 × 10^6^ cells.

### Statistical analysis

All data are expressed as the means ± SD from three independent experiments. Statistical analysis was performed using the STATISTICA version 12.5 software (Statsoft, Inc., Tulsa, OK, USA). One-way analysis of variance (ANOVA) followed by Tukey’s post hoc test were used to determine the differences between the mean values of multiple groups. The equality of variances assumption was verified with the Levene’s test. Statistical significance was considered at p<0.05.

## Results

### Berry fruit extract composition

**Fig 1:**
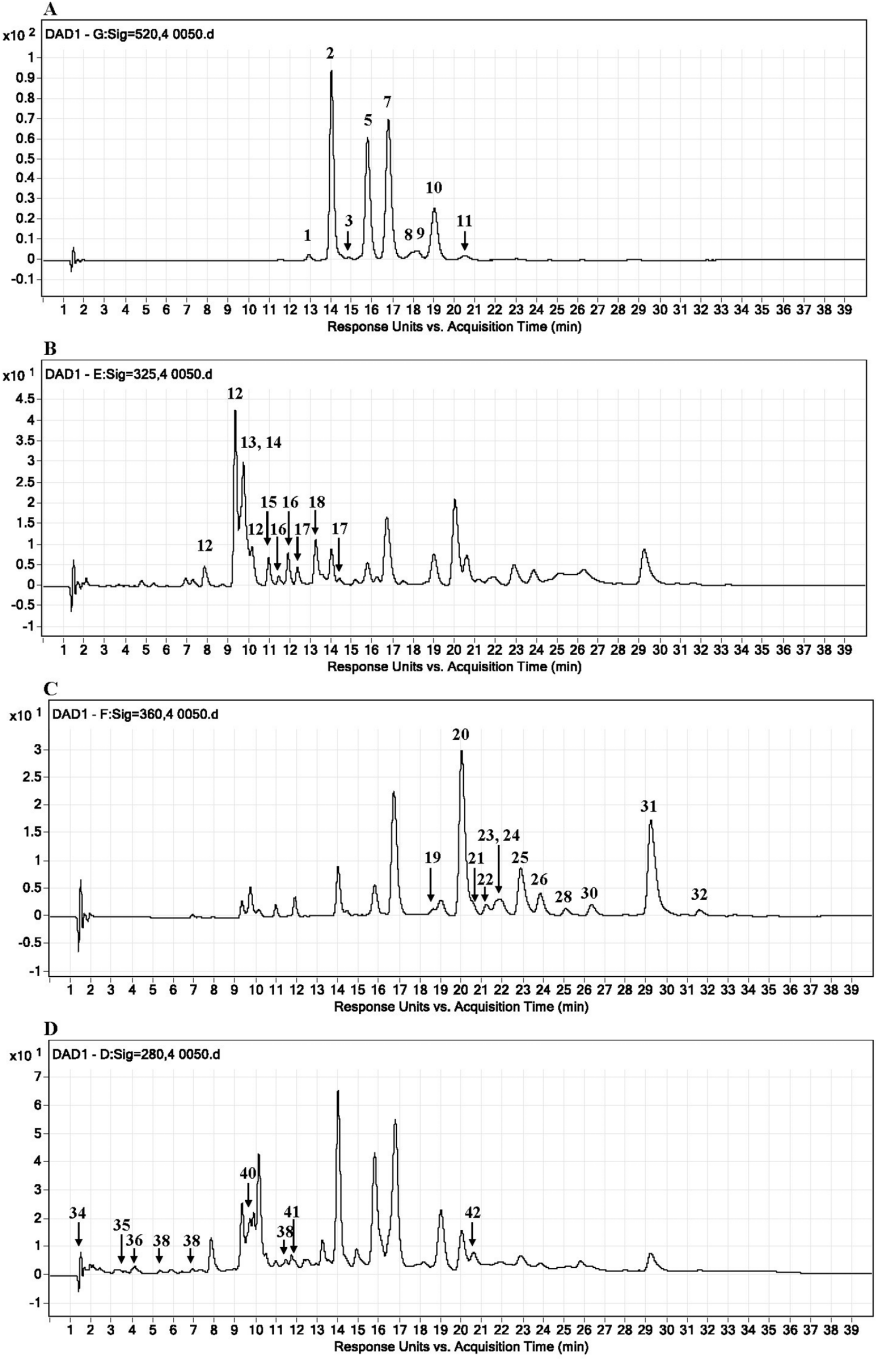
HPLC-DAD chromatograms of the berry fruit extract. HPLC-DAD traces at 520 (A), 325 (B), 360 (C) and 280 (D) nm, respectively (chromatograms recorded after 80-fold dilution of the berry fruit extract). Peak numbers correspond to the compounds listed in Table 1.

The results of the HPLC-DAD-ESI-MS analysis of polyphenol compounds tentatively identified in the extract of berry fruits are shown in Table 1. The HPLC-DAD chromatograms obtained for the extract at four wavelengths are presented in Fig 1A–1D.

**Table 1:** Retention times (t_R_), mass spectral data, tentative identification and phenolic compounds quantification (μg/mL) in the extract prepared from berry fruits.

Peak No.	UV detection t_R_ (min)	MS detection t_R_ (min)	Precursor ion *m/z*	Ioni-zation mode	Major fragment ion (*m*/*z*)	Chemical formula	Tentative identification	Concentration (μg/mL)
**1**	**2**	**3**	**4**	**5**	**6**	**7**	**8**	**9**
**ANTHOCYANINS**								
1*	12.88	13.05	465.1011	(+)	303.0484	C_21_H_21_O_12_	delphinidin-3-*O*-galactoside	231.0 ± 9.6
2*	13.97	14.12	449.1130	(+)	287.0633	C_21_H_21_O_11_	cyanidin-3-*O*-galactoside	7429.1 ± 151.5
3*	14.82	15.02	449.1124	(+)	287.0583	C_21_H_21_O_11_	cyanidin-3-*O*-glucoside	116.5 ± 7.1
4#	15.44	15.60	479.1205	(+)	317.0751	C_22_H_23_O_12_	petunidin-3-*O*-galactoside	119.5 ± 6.6
5*	15.74	15.88	419.1020	(+)	287.0607	C_20_H_19_O_10_	cyanidin-3-*O*-arabinoside	5810.0 ± 142.8
6#^	—	16.24	433.1135	(+)	271.0596	C_21_H_21_O_10_	pelargonidin 3-*O*-glucoside	—
7*	16.75	16.85	463.1220	(+)	301.0705	C_22_H_22_O_11_	peonidin-3-*O*-galactoside	7358.3 ± 137.2
8*	17.95	18.07	463.1286	(+)	301.0753	C_22_H_22_O_11_	peonidin-3-*O*-glucoside	259.0 ± 12.7
9*	18.13	18.29	433.1157	(+)	301.0702	C_21_H_21_O_10_	peonidin-3-*O*- arabinoside	502.4 ± 20.7
10*	18.97	19.12	493.1351	(+)	331.0843	C_23_H_25_O_12_	malvidin-3-*O*-galactoside	3312.6 ± 129.9
11*	20.47	20.69	419.1014	(+)	287.0584	C_20_H_19_O_10_	cyanidin-3-*O*-xyloside	304.7 ± 15.8
**HYDROXYCINNAMIC ACID DERIVATIVES**								
**1**	**2**	**3**	**4**	**5**	**6**	**7**	**8**	**9**
12*	7.81 9.30 9.87 10.10	7.94 9.43 10.11 10.24	325.0912 325.0929 325.0908 325.0929	(-)	163.0389 145.0283 163.0401 145.0295	C_15_H_18_O_8_	*p*-coumaric acid-glucoside	417.1 ± 12.2 2270.7 ± 145.9 524.4 ± 25.6 696.1 ± 28.2
13*	9.59	9.72	341.0870	(-)	179.0337	C_15_H_18_O_9_	caffeoylglucose	617.0 ± 25.1
14*	9.69	9.87	353.0874	(-)	191.0568	C_16_H_18_O_9_	chlorogenic acid	1517.6 ± 78.5
15*	10.94	11.11	355.1031	(-)	193.0500	C_16_H_20_O_9_	ferulic-β-glucoside	468.4 ± 20.4
16*	11.42 11.88	11.54 12.02	385.1622 385.1620	(-)	223.0988 223.0963	C_17_H_22_O_10_	sinapoylglucose	244.6 ± 10.2 527.0 ± 18.0
17*	12.33 14.36	12.48 14.55	337.0917 337.0929	(-)	173.0443 173.0455	C_16_H_18_O_8_	*p*-coumaroylquinic acid	392.0 ± 15.9 223.3 ± 14.1
18*	13.21	13.42	371.3016	(-)	163.0396	—	*p*-coumaric acid derivative	968.3 ± 28.5
**FLAVONOLS**								
**1**	**2**	**3**	**4**	**5**	**6**	**7**	**8**	**9**
19*	18.59	18.71	449.0726	(-)	317.0276	C_20_H_18_O_12_	myricetin 3-*O*-arabinoside	9.3 ± 0.6
20*	19.97	20.10	463.0874	(-)	301.0334	C_21_H_20_O_12_	quercetin-3-*O*-galactoside	621.8 ± 23.5
21*	20.49	20.67	463.0882	(-)	301.0354	C_21_H_20_O_12_	quercetin-3-*O*-glucoside	23.9 ± 2.0
22*	21.17	21.37	609.1461	(-)	301.0349	C_27_H_30_O_16_	quercetin 3-*O*-rutinoside	33.5 ± 2.2
23*	21.72	21.83	493.0970	(-)	331.0437	C_22_H_22_O_13_	laricitrin-3-*O*-glu/gal	34.9 ± 2.8
24*	21.85	22.11	433.0786	(-)	301.0324	C_20_H_18_O_11_	quercetin-3-*O*-arabinoside	63.7 ± 4.1
25*	22.85	23.03	317.0284	(-)	—	C_15_H_10_O_8_	myricetin	210.5 ± 10.2
26*	23.79	23.98	447.0935	(-)	301.0349	C_21_H_20_O_11_	quercetin-3-*O*-rhamnoside	88.6 ± 4.6
27#^	—	24.73	447.0935	(-)	285.0540	C_21_H_20_O_11_	kaempferol-3-*O*-glucoside	—
28*	25.03	25.21	477.1039	(-)	315.0511	C_22_H_22_O_12_	3′-methoxy-quercetin-3-*O*-galactoside	18.2 ± 1.5
29#^	—	25.30	593.1515	(-)	285.0542	C_27_H_30_O_15_	kaempferol-3-*O*-rutinoside	—
30*	26.28	26.46	507.3841	(-)	463.3342	C_23_H_24_O_13_	dimethoxymyricetin-hexoside	39.2 ± 2.1
31*	29.17	29.31	301.0356	(-)	151.0031	C_15_H_10_O_7_	quercetin	463.7 ± 15.1
32*	31.52	31.69	514.5579	(-)	331.1308	—	laricitrin derivative	17.6 ± 1.3
33#^	—	33.44	567.1117	(-)	301.0329	C_28_H_24_O_13_	quercetin-3-O-(6”benzoyl)-*β*-galactoside	—
**HYDROXYBENZOIC ACID DERIVATIVES AND OTHERS**								
**1**	**2**	**3**	**4**	**5**	**6**	**7**	**8**	**9**
34*	1.46	1.62	191.0567	(-)	173.0444	C_7_H_12_O_6_	quinic acid	165.8 ± 8.0
35#	3.65	3.80	779.2248	(-)	389.1082	C_32_H_44_O_22_	monotropein	28.2 ± 2.0
36*	4.09	4.23	169.0142	(-)	125.0242	C_7_H_6_O_5_	gallic acid	111.3 ± 7.5
37#	4.74	4.89	315.0702	(-)	153.0183	C_13_H_16_O_9_	protocatechuic-acid-4-glucoside	38.4 ± 3.1
38#	5.31 6.89 11.27	5.33 7.09 11.40	467.1185 467.1176 467.1188	(-)	305.0657 305.0659 305.0653	C_21_H_24_O_12_	epigallocatechin 3-glu/gal or gallocatechin 3-glu/gal	43.7 ± 3.5 35.2 ± 3.1 32.1 ± 3.0
39#	5.84	5.99	153.1504	(-)	109.0284	C_7_H_6_O_4_	3,4-dihydroxy-benzoic acid/ protocatechuic acid	44.5 ± 3.4
40*	9.39	9.50	577.1340	(-)	407.0763	C_30_H_26_O_12_	procyanidin B1 or B2	146.9 ± 9.9
41*	11.71	11.86	289.0719	(-)	245.0799	C_15_H_14_O_6_	catechin or epicatechin	218.8 ± 12.7
42*	20.55	21.08	435.1291	(-)	273.0755	C_21_H_24_O_10_	phlorizin	293.6 ± 14.2

* number corresponds to the peak shown in Fig 1,

^#^ trace amounts,

^ identified only by MS detection,

− value not determined.

The results of the HPLC-based quantitative analysis are expressed as the means ± SD, n = 3.

It was established that the extract contained many different polyphenol compounds, including anthocyanins, as a major group of polyphenols. LC-ESI-MS analysis of the extract in positive ion mode allowed the identification of eleven anthocyanin derivatives including four major glycosylated derivatives of cyanidin (cyanidin-3-*O*-galactoside and cyanidin-3-*O*-arabinoside), peonidin (peonidin-3-*O*-galactoside), and malvidin (malvidin-3-*O*-galactoside) (peaks 2, 5, 7 and 10, respectively; Table 1, Fig 1A). These derivatives were dominant and constituted approx. 94% of all of the anthocyanin compounds, with a significant predominance of 3-*O*-galactosides (75.7%) (Table 1). In addition, the 3-*O*-glucoside and 3-*O*-arabinoside of peonidin (peaks 8 and 9), the 3-*O*-galactoside of delphinidin and petunidin (peaks 1 and 4) and the glycosides of cyanidin (cyanidin-3-*O*-glucoside and cyanidin-3-*O*-xyloside) (peaks 3 and 11) were identified and quantified in a minor amount in the BFE (Table 1, Fig 1A). The presence of pelargonidin-3-*O*-glucoside in the extract was detected only by MS analysis (Table 1). The total amount of anthocyanin derivatives was estimated at 25.4 ± 0.6 mg cyanidin-3-*O*-galactoside/mL, which accounted for 68.6% of all polyphenolic compounds identified in the berry fruit extract.

Other groups of polyphenolic compounds: hydroxycinnamic acid derivatives and flavonols amounted to 23.9% and 4.4% of the total content of polyphenols, respectively. A total of 7 compounds were detected within the group of hydroxycinnamic acid derivatives, including chlorogenic acid (17.1%), *p*-coumaroylquinic acid (6.9%) and derivatives of *p*-coumaric acid (55%), ferulic acid (5.3%), caffeic acid (7.0%) and sinapic acid (8.7%). The content of these compounds in the extract was determined at 8.9 ± 0.4 mg/mL (Table 1). With regard to flavonols, the results of the HPLC-DAD analysis revealed the presence of derivatives of quercetin, myricetin, kaempferol, and laricitrin in a total amount of 1.6 ± 0.1 mg/mL, with a predominance of quercetin and its glycosides (81%) (Table 1).

Other groups of compounds, including hydroxybenzoic acid derivatives, were estimated at 1.2 ± 0.1 mg/mL, which accounted for 3.1% of all quantified polyphenols in the extract (Table 1).

### The effects of berry fruit extract on adipogenesis, lipogenesis, and intracellular ROS production

In this experiment, adipocytes undergoing induced differentiation were treated with various concentrations of BFE (25, 50 and 100 μg/mL). Cell viability, metabolic activity, cell oxidation status, and lipid accumulation were analyzed in the culture after the complete differentiation process.

**Fig 2:**
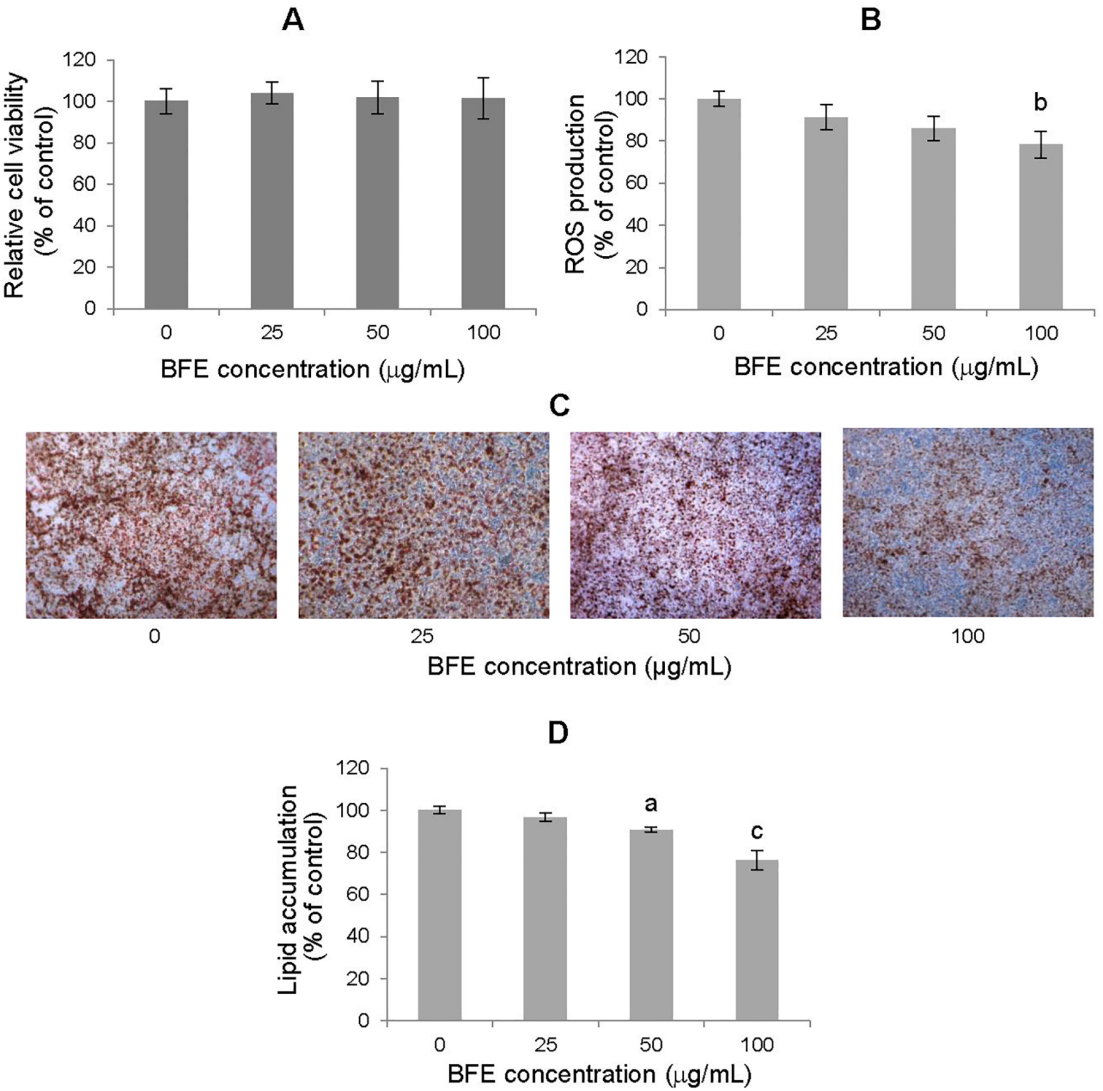
Effects of berry fruit extract (BFE) on adipogenesis induced in 3T3-L1 cells. The effect of BFE on the viability (A), ROS production (B) and lipids accumulation (C–D) in 3T3-L1 adipocytes undergo the differentiation process. Microscopic images of adipocytes stained with Oil Red O (C) and the fat content measured after Oil Red O elution (D). Extract was added to the 3T3-L1 cell cultures at each stage of differentiation process at concentrations of 25, 50 and 100 μg/mL. Data are the mean values ± SD (n = 3). The results of a one-way ANOVA indicated significant effects of the extract on lipid concentration (*F* = 45.4, *p* < 0.0001) and ROS accumulation (*F* = 8.16, *p* < 0.0082) in differentiated fat cells. ^a^p < 0.05, ^b^p < 0.01, ^c^p < 0.001 (Tukey’s post hoc test).

The MTT results showed that the introduction of BFE into the 3T3-L1 cell culture during differentiation did not affect cell viability or metabolic activity, showing no cytotoxic effect on adipocytes (Fig 2A). Staining lipid droplets with Oil Red *O* indicated that the lipid accumulation in cells that were exposed to the extract was dose-dependent (*F* = 45.4, *p* < 0.0001) (Fig 2C and 2D). The intracellular lipid content was reduced by 23.7% following treatment with the extract at a concentration of 100 μg/mL (*p* = 0.0002).

The introduction of BFE at the highest dose of 100 μg/mL caused a reduction in the ROS level by 21.8% compared with the control adipocytes that were not treated with the extract (*p* = 0.0061) (Fig 2B).

### The effect of berry fruit extract on expression of genes associated with adipogenesis and lipogenesis

**Fig 3:**
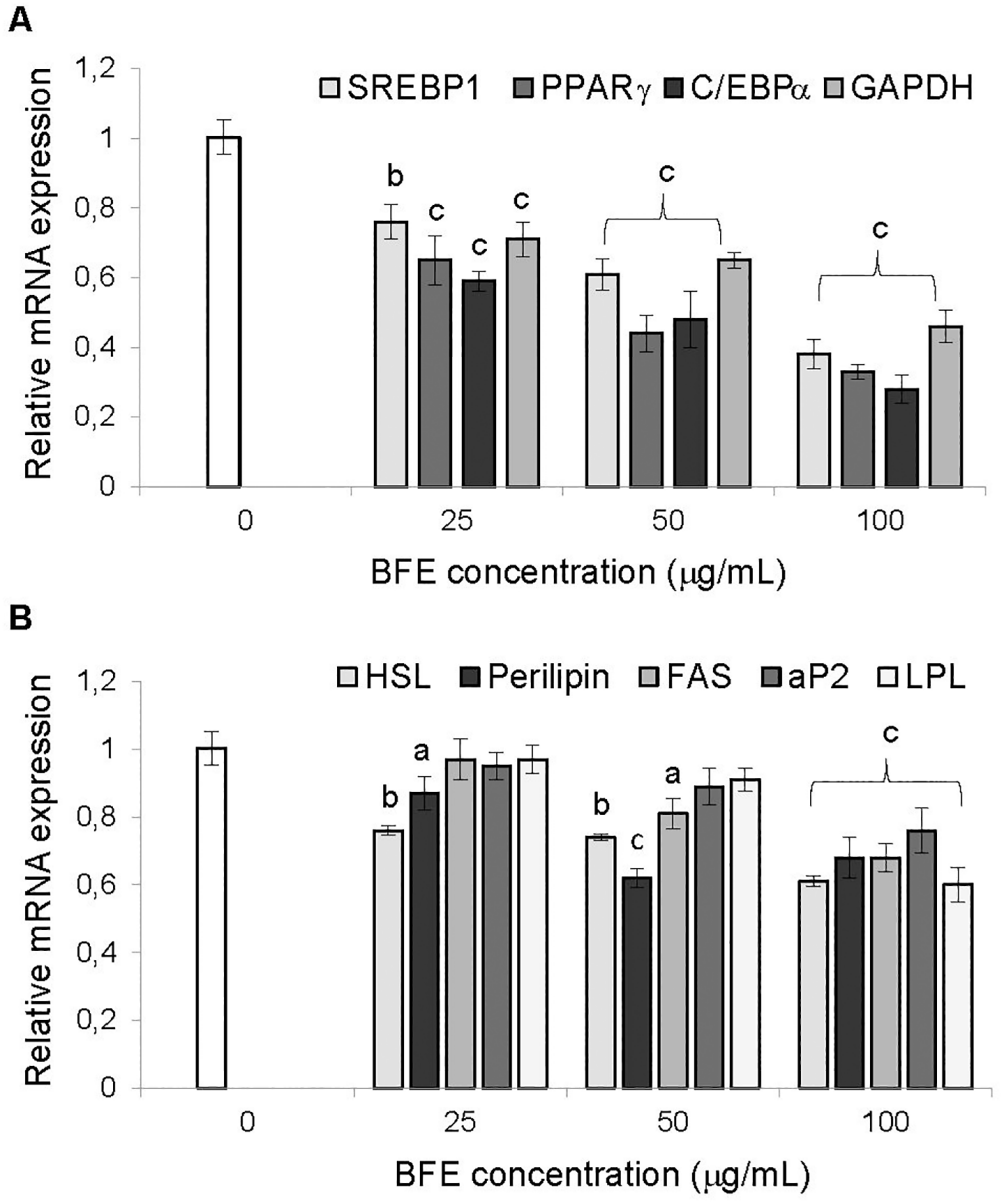
Effect of the extract on the expression of genes associated with adipogenesis (A) and lipogenesis (B). The berry fruit extract (BFE) was added to the 3T3-L1 cell cultures at each stage of differentiation process at concentrations of 25, 50 and 100 μg/mL. The expression level of each gene was quantified by real-time PCR and normalized using *β-actin* as an internal control. Data are the mean values ± SD (n = 3). ^a^*p* < 0.05, ^b^*p* < 0.01, ^c^*p* < 0.001 (Tukey’s post hoc test).

**Fig 4:**
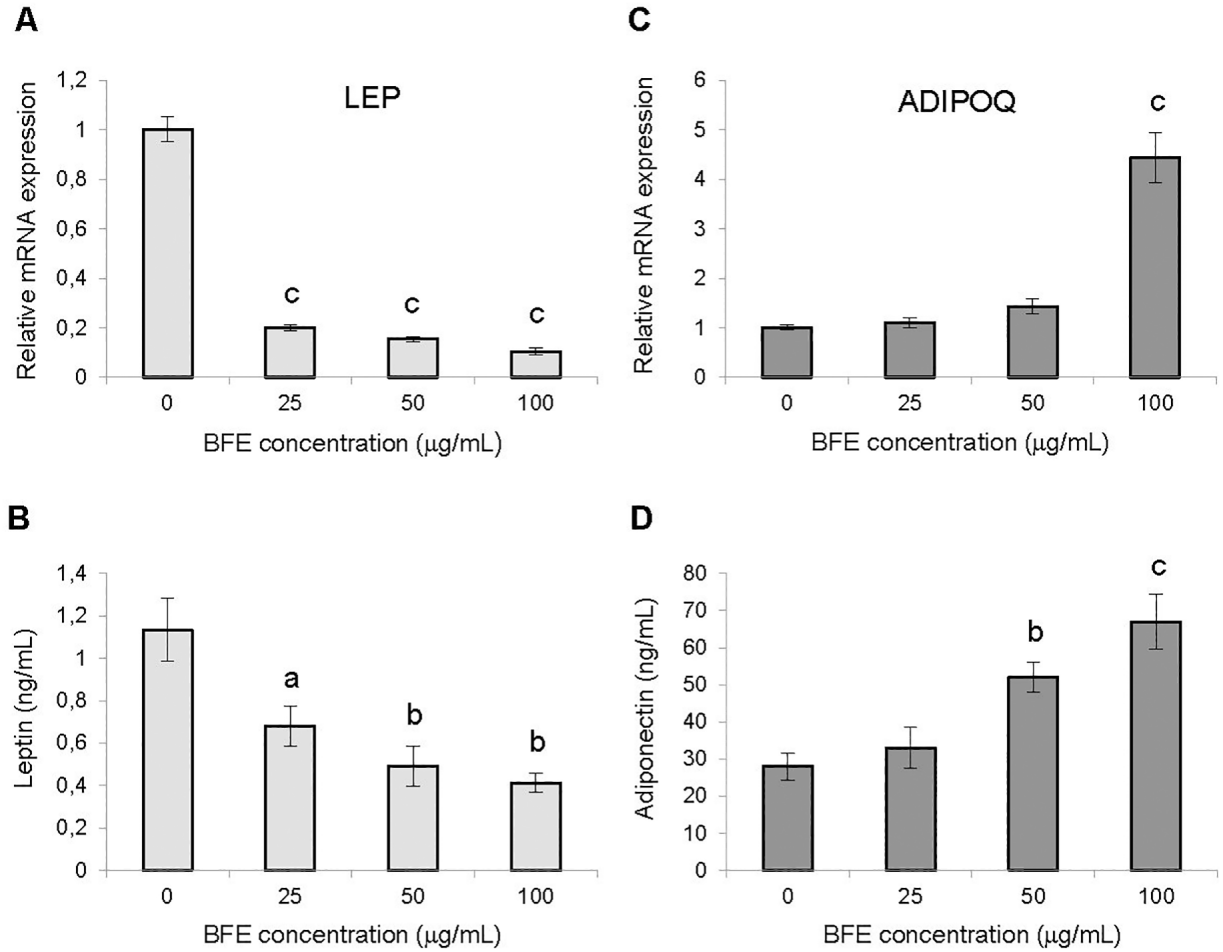
Effect of the extract on mRNA expression of leptin (A) and adiponectin (B), and adipokine secretion (C–D). The berry fruit extract (BFE) was added to the 3T3-L1 cell cultures at each stage of differentiation process at concentrations of 25, 50 and 100 μg/mL. The expression levels of *LEP* and *ADIPOQ* genes were quantified by real-time PCR and normalized using β-actin as an internal control. Leptin and adiponectin concentrations were determined in the cell culture supernatants. Data are the mean values ± SD (n = 3). ^a^*p* < 0.05, ^b^*p* < 0.01, ^c^*p* < 0.001 (Tukey’s post hoc test).

To elucidate the molecular mechanisms for reducing the lipid content in adipocytes caused by BFE, the influence that the extract had on the expression of genes involved in adipogenesis and lipogenesis was investigated. Quantitative PCR analysis revealed that BFE treatment significantly inhibited peroxisome proliferator-activated receptor gamma (*PPARγ*), CCAAT/enhancer-binding protein alpha (*C/EBPα*), sterol regulatory element binding transcription factor 1 (*SREBP1*), adipocyte fatty acid-binding protein (*aP2*), fatty acid synthase (*FAS*), lipoprotein lipase (*LPL*), hormone-sensitive lipase (*HSL*), perilipin 1 (*PLIN1*) and glyceraldehyde-3-phosphate dehydrogenase (*GAPDH*) mRNA expression in a dose-dependent manner (Fig 3A and 3B). At the highest dose (100 μg/mL), BFE inhibited the expression of *PPARγ*, *C/EBPα*, *SREBP1* and *GAPDH* genes by 67, 72, 62 and 54%, respectively (*p* < 0.001). Exposure of differentiating 3T3-L1 cells to the extract down-regulated leptin (*LEP*) mRNA expression compared to adipocytes that differentiated without BFE treatment (Fig 4A). Real-time PCR analysis revealed that BFE substantially inhibited *LEP* expression, with a significant 90, 85 and 80% decrease at a dose of 100, 50 and 25 μg/mL, respectively, compared to untreated cells (*p* < 0.001).

In contrast, BFE had an enhancing effect on adiponectin gene (*ADIPOQ*) expression (Fig 4B). At all tested doses, the expression of adiponectin was up-regulated, with a significant increase at the highest dose of 100 μg/mL, where *ADIPOQ* expression increased by 4.4-fold compared to untreated adipocytes (*p* < 0.001).

### The effect of berry fruit extract on adipokine secretion

Treating differentiating 3T3-L1 cells with BFE significantly enhanced adiponectin secretion in a dose-dependent manner (*F* = 47.8, *p* < 0.0001). Supplementation of 3T3-L1 cell culture with BFE at doses of 25, 50 and 100 μg/mL led to increases of 7.1, 78.6 and 166.8% in adiponectin secretion, respectively (Fig 4D). However, BFE treatment induced a dose-dependent decrease in leptin concentration (*F* = 29.6, *p* = 0.0001). A reduction of leptin secretion by 56.6 and 63.7% was obtained in the 3T3-L1 cells treated with BFE at doses of 50 and 100 μg/mL, respectively (Fig 4C).

## Discussion

Adipocyte differentiation is a highly controlled, multi-step process that involves a cascade of transcription factors and cell-cycle proteins that regulate gene expression and lead to adipocyte development. PPARγ and C/EBPα are critical transcription factors in adipogenesis. However, C/EBPα is incapable of inducing adipogenesis in the absence of PPARγ [14]. Overexpression of PPARγ in mature 3T3-L1 adipocytes increases both cell size and the intracellular content of triglycerides [15]. Therefore, modulation of PPARγ activity by functional food compounds may be a complementary treatment for obesity-related disorders [2]. Additionally, C/EBPα is considered an important factor in the development of adipose tissue, which directly induces many adipocyte genes. SREBP1c mediates the induction of lipid biosynthesis in adipocytes by increasing the gene expression of the main lipogenic genes [16]. The increased activity and the protein and mRNA levels of the enzymes involved in triacylglycerol synthesis and degradation, including FAS, GAPDH, aP2, a fatty acid transporter, and PLIN1, a lipid droplet-associated protein, are specific to the terminal phase of differentiation. The synthesis of adipocyte-secreted products, including adiponectin, leptin and other cytokines, also begins during the late phase of differentiation [17].

The effects of BFE prepared from chokeberry, raspberry, bilberry and cranberry fruits on the expression of genes involved in adipogenesis and lipogenesis were evaluated in this study. Quantitative PCR analysis revealed that BFE treatment inhibited *PPARγ*, *C/EBPα*, *SREBP1*, *aP2*, *FAS*, *LPL*, *HSL* and *PLIN1* mRNA expression in a dose-dependent manner, which resulted in decreased intracellular lipid accumulation. BFE also significantly down-regulated *LEP* mRNA expression and leptin secretion in differentiated adipocytes, which may have beneficial effects on leptin resistance. The level of leptin is closely associated with the amount of stored body fat and the body mass index [18], and it has been shown to have direct pro-inflammatory and catabolic effects [19]. In contrast to leptin, the level of adiponectin secreted by adipocytes differentiating in the presence of BFE was significantly up-regulated in a dose-dependent manner. The increased adiponectin production caused by BFE can be associated with a decreased level of lipids accumulating in adipocytes and a decline in leptin secretion. Since adiponectin is almost exclusively secreted by adipocytes and appears to act as a hormone with anti-inflammatory and insulin-sensitizing properties [20], the analyzed extract could have the ability to act as an insulin-sensitizing agent and could be a valuable supplement for both obese and non-obese patients suffering from insulin resistance.

Berries are a rich source of antioxidants and phytochemicals, including flavonoids (anthocyanins, flavonols and flavanols), tannins (proanthocyanidins, ellagitannins, and gallotannins), stilbenoids, and phenolic acids, which have received considerable interest due to their possible relations to human health [9, 21]. The anti-obesity mechanisms for berries may include a reduction in lipid absorption, a decrease in differentiation and proliferation of preadipocytes, a decrease in lipogenesis, an increase in lipolysis, and an inhibition of pro-inflammatory adipokine secretion [10, 11]. Raspberry and bilberry were found to diminish lipid accumulation with a concomitant down-regulation of *PPARγ*, *C/EBPα* and *SREBP1c* in 3T3-L1 adipocytes and to suppress the expression of *aP2* and *resistin* [8, 9]. Cranberries decreased the number of adipocytes and reduced lipid accumulation in maturing 3T3-L1 preadipocytes, demonstrating an inhibitory effect on lipogenesis. It was found that cranberries directly induced lipolysis in adipocytes and down-regulated the expression of major transcription factors of the adipogenesis pathway such as *PPARγ*, *C/EBPα*, and *SREBP1* [11]. The reduced lipid accumulation during preadipocyte differentiation was through down-regulation of *aP2*, *FAS*, *LPL*, *HSL*, and *PLIN1* gene expression. Moreover, cranberries decreased leptin and increased adiponectin gene expression and protein secretion in 3T3-L1 cells [10]. Likewise, chokeberry inhibited the protein and gene expression of *aP2*, *FAS* and *LPL* [22]. In obese animals, chokeberry suppressed visceral fat accumulation and hyperglycemia, elevated plasma adiponectin and inhibited the plasma TNF-α and IL6 levels [23]. However, doses of extracts obtained from individual raspberry, bilberry, chokeberry, and cranberry fruits were significantly higher than doses of multicomponent berry polyphenol extract which were sufficient to produce beneficial anti-obesity effects.

The results obtained from few studies indicate that anti-obesity effects of berry polyphenols may be potentiated in multicomponent phenolic extracts. Multiple phenolic-rich extract of the Alaskan berries *Vaccinium ovalifolium*, *Vaccinium uliginosum*, *Empetrum nigrum*, *Rubus spectabilis*, and *Rubus chamaemorus* decreased the lipid levels within mature adipocytes and increased the expression of *PREF-1*, preadipocyte secreted factor 1, which inhibits adipogenesis [7]. Interestingly, anti-lipogenic activity was lost when the extract was fractionated and component phytochemicals were administered separately. This result suggests that interactions between phytochemicals in the mixed extract are required to potentiate biological activity.

The analyzed BFE, prepared from chokeberry, raspberry, bilberry and cranberry fruits, contained many different polyphenol compounds, among which the anthocyanins constituted the major group of BFE polyphenols. Cyanidin- and peonidin-based derivatives were the main anthocyanin compounds. The anti-obesity properties of anthocyanins have been investigated extensively. The first report to demonstrate the preventive potential of anthocyanins against body fat accumulation was published by Tsuda and colleagues [24]. In this study, C57BL/6J mice supplemented with cyanidin-3-*O*-glucoside significantly suppressed the body fat accumulation induced by a high-fat diet [24]. The same authors showed that anthocyanins act on adipose tissue by inducing changes in the expression of adipokines in isolated rat and human adipocytes. They reported that cyanidin-3-*O*-glucoside and its aglycone induce the up-regulation of adiponectin, which enhances insulin sensitivity [25, 26]. In 3T3-L1 cells, anthocyanins reduced lipid accumulation and suppressed the expression of *PPARγ* [27]. Moreover, hydroxycinnamic acid derivatives and flavonols, mainly quercetin and myricetin, which constitute BFE components, may induce positive effects in the treatment or prevention of diabetes, obesity, and other age- and metabolism-related diseases [28]. In the experimental studies on differentiating preadipocytes, quercetin decreased the expression of *LPL*, *SREBP1c*, and *PPARγ*, a key adipogenic transcription factor [29]. Myricetin inhibited the differentiation of 3T3-L1 preadipocytes and down-regulated the mRNA and protein levels of C/EBPα PPARγ, SREBP1, aP2, LPL and perilipin A [30]. Hydroxycinnamic acid derivatives also showed therapeutic potential in experimental diabetes and hyperlipidemia. These compounds inhibited macrophage infiltration, reduced the expression of potent pro-inflammatory adipokines in obese animals, and increased the secretion of adiponectin by adipocytes. Furthermore, hydroxycinnamic acid derivatives prevented adipocyte differentiation and lowered the lipid profile in experimental animals [31].

There is strong evidence that the whole plant extracts and the mixtures of flavonoids exert synergistic and superior effects compared to the isolated phytochemicals. In 3T3-L1 adipocytes, adipogenesis was substantially inhibited by a *Hibiscus sabdariffa* extract, and the effect of the full extract was higher than the sum of its fractions. These studies provided further evidence that a combination of bioactive components was superior to isolated constituents [32]. Although some isolated phenolic compounds such as quercetin or anthocyanins have also shown the ability to inhibit adipogenesis in 3T3-L1 cell culture, a cytotoxic effect on adipocytes was observed [27, 29]. Plant-derived polyphenols are a complex mixture that interact with numerous endogenous molecular targets in humans but are surprisingly safe even at high doses. Thus, phytotherapy, whose therapeutic efficacy is based on the combined action of a mixture of constituents, offers valuable treatment opportunities [3].

## Conclusions

To summarize the results of this study, we found that the multicomponent polyphenol-rich extract obtained from chokeberry, raspberry, bilberry, and cranberry can have a beneficial effect on the prevention or treatment of obesity via the inhibition of adipogenesis, lipid accumulation and ROS production in adipocytes without affecting their viability. In the future, in vivo studies will be undertaken to confirm the anti-obesity potential of the extract found in the experiments performed using in vitro adipocyte 3T3-L1 model.

## Supporting information

S1 Table The primers sequence used for real-time PCR. (PDF)
