# Supplementary material for: Inhibitory activity of chokeberry, bilberry, raspberry and cranberry polyphenol-rich extract towards adipogenesis and oxidative stress in differentiated 3T3-L1 adipose cells
Source: PLoS One. 2017 Nov 28;12(11):e0188583. doi: 10.1371/journal.pone.0188583 (PMC5705115; doi:10.1371/journal.pone.0188583)
Supplement: S1 Table — (PDF) [file pone.0188583.s001.pdf]

Table S1. The primers sequence used for real-time PCR.

Gene	Accession	No. Sequence (5' – 3')	Amplicon (bp)
PPARγ	NM-011146	F: TTT TCA AGG GTG CCA GTT TC<br>R: AAT CCT TGG CCC TCT GAG AT	198
C/EBPα	NM-007678	F: TTA CAA CAG GCC AGG TTT CC<br>R: GGC TGG CGA CAT ACA GTA CA	188
SREBP1	NM-011480	F: TGT TGG CAT CCT GCT ATC TG<br>R: AGG GAAAGC TTT GGG GTC TA	190
GAPDH	NM-008084	F: AATGAAGGGGTCGTTGATGG<br>R: ATGGTGAAGGTCGGTGTGAA	108
LPL	NM-008509	F: TCC AAG GAA GCC TTT GAG AA<br>R: CCA TCC TCA GTC CCA GAA AA	188
FAS	NM-007988	F: TTG CTG GCA CTA CAG AAT GC<br>R: AAC AGC CTC AGA GCG ACA AT	192
PLIN1	NM-175640	F: AAG GAT CCT GCA CCT CAC AC<br>R: CCT CTG CTG AAG GGT TAT CG	191
HSL	NM-010719	F: GGA CAC ACA CACACC TG<br>R: CCC TTT CGC AGC AAC TTT AG	190
LEP	NM-008493	F: GGA TCA GGT TTT GTG GTG CT<br>R: TTG TGG CCC ATA AAG TCC TC	187
FABP4 (aP2)	NM-024406	F: TCA CCT GGA AGA CAG CTC CT<br>R: AAT CCC CAT TTA CGC TGA TG	182
ADIPOQ	NM-009605	F: CTGGCCACTTTCTCCTCATTTC<br>R: GGCATGACTGGGCAGGATTA	120
ACTB	NM-007393	F: CCA CAG CTG AGA GGG AAA TC<br>R: AAG GAA GGC TGG AAA AGA GC	193
